# Whole-Genome Sequence of *Microcystis aeruginosa* TAIHU98, a Nontoxic Bloom-Forming Strain Isolated from Taihu Lake, China

**DOI:** 10.1128/genomeA.00333-13

**Published:** 2013-06-13

**Authors:** Chen Yang, Wei Zhang, Minglei Ren, Lirong Song, Tao Li, Jindong Zhao

**Affiliations:** Key Laboratory of Algal Biology, Institute of Hydrobiology, Chinese Academy of Science, Wuhan, Chinaa; College of Life Science, Peking University, Beijing, Chinab

## Abstract

*Microcystis aeruginosa* is a dominant bloom-forming cyanobacterium in many freshwater lakes. This report describes the first whole-genome sequence of the nontoxic strain of *M. aeruginosa* TAIHU98, which was isolated from Taihu Lake in eastern China.

## GENOME ANNOUNCEMENT

*Microcystis aeruginosa* is one of the most ecologically harmful and dominant bloom-forming cyanobacteria in freshwater lakes (1). The production of microcystin, a cyclic heptapeptide made by *M. aeruginosa* and some other toxic cyanobacteria, is now a major concern for water safety (2). Also, the accumulation of *M. aeruginosa* mass on the surface water has deteriorative effects on freshwater ecosystems, such as blocking light for other photosynthetic organisms and causing hypoxia in the water. In many freshwater lakes, such as Taihu Lake in eastern China, *Microcystis* water bloom becomes dominant in the summer and lasts until early winter (3, 4). Despite the concentration of microcystin being highest in the summer in Taihu Lake (5), toxic and nontoxic cells of *M. aeruginosa* coexist during the entire period of the water bloom (6). To understand the mechanism of water-bloom formation and its ecological effects, we isolated both toxic and nontoxic *M. aeruginosa* strains from Taihu Lake water bloom. Here, we report the whole-genome sequence of the nontoxic *M. aeruginosa* strain TAIHU98.

Whole-genome sequencing of TAIHU98 was performed with a combination of Genome Sequencer FLX (Roche) (400-bp single-end library and 3-kb paired-end library, 1,070,319 reads) and Genome Analyzer IIx (Illumina) (3-kb mate-pair library, 6,867,274 reads). All 454 reads were assembled into 395 contigs by Newbler v2.0.01.14. Paired-end and Solexa mate-pair reads were used to order these contigs into 50 supercontigs within 6 scaffolds. Gaps were then filled by sequencing PCR products with the help of Phred-Phrap-Consed v23.0. The prediction of protein-encoding sequences (CDSs) was generated by Glimmer 3.0 (7) and GeneMark 2.5 (8). tRNAs and rRNAs were detected using tRNAscan-SE 1.23 (9) and RNAmmer 1.2 (10), respectively. Functional annotation of the genome was performed by searching against NCBI nonredundant protein, InterPro, and COG databases (11).

The genome of TAIHU98 comprises 4 supercontigs with a total length of 4,849,611 bp and an average G+C content of 42.45%. It contains 5,356 protein-coding genes and 48 genes coding for RNA (two sets of rRNA genes and 42 tRNA genes). According to annotation results, 2,660 putative genes show similarity to the genes with known functions, and the remaining 2,696 genes were determined as encoding hypothetical proteins or were assigned putative functions. A total of 349 copies of insertion sequence (IS) transposase genes are assigned to 19 families, confirming that the genome was as highly plastic as *M. aeruginosa* strains NIES843 (12) and PCC7806 (13).

Comparative analysis revealed that the nucleic acid base sequence of TAIHU98 bears a similarity to those of *M. aeruginosa* PCC7806 (76.5%) and *M. aeruginosa* NIES843 (64.58%). The three genomes only share 2,511 CDSs involved in cell structure components and primary metabolism processes, while TAIHU98 has 1,559 strain-specific genes, mainly with unknown functions. This large variation in the genomes indicates that each strain has gained a considerable number of genes during evolution.

The TAIHU98 genome is missing all microcystin synthetase (*mcy*) genes (14) and cyanopeptolin synthetase (*mcn*) genes (15), while the whole aeruginosin synthetase (*aer*) gene cluster (16) is present.

### Nucleotide sequence accession numbers.

This Whole-Genome Shotgun project has been deposited at DDBJ/EMBL/GenBank under the accession no. ANKQ00000000. The version described in this paper is the first version, accession no. ANKQ01000000 .
